# Structural Basis of Ca^2+^-Dependent Self-Processing Activity of Repeat-in-Toxin Proteins

**DOI:** 10.1128/mBio.00226-20

**Published:** 2020-03-17

**Authors:** Vojtech Kuban, Pavel Macek, Jozef Hritz, Katerina Nechvatalova, Katerina Nedbalcova, Martin Faldyna, Peter Sebo, Lukas Zidek, Ladislav Bumba

**Affiliations:** a Central European Institute of Technology, Masaryk University, Brno, Czech Republic; b National Centre for Biomolecular Research, Faculty of Science, Masaryk University, Brno, Czech Republic; c Celonic AG, Basel, Switzerland; d Department of Immunology, Veterinary Research Institute, Brno, Czech Republic; e Laboratory of Molecular Biology of Bacterial Pathogens, Institute of Microbiology, Academy of Sciences of the Czech Republic, Prague, Czech Republic; Institut Pasteur

**Keywords:** RTX toxins, cell adhesion, clip-and-link, host-pathogen interactions, nuclear magnetic resonance

## Abstract

The posttranslational Ca^2+^-dependent “clip-and-link” activity of large repeat-in-toxin (RTX) proteins starts by Ca^2+^-dependent structural rearrangement of a highly conserved self-processing module (SPM). Subsequently, an internal aspartate-proline (Asp-Pro) peptide bond at the N-terminal end of SPM breaks, and the liberated C-terminal aspartyl residue can react with a free ε-amino group of an adjacent lysine residue to form a new isopeptide bond. Here, we report a solution structure of the calcium-loaded SPM (Ca-SPM) derived from the FrpC protein of Neisseria meningitidis. The Ca-SPM structure defines a unique protein architecture and provides structural insight into the autocatalytic cleavage of the Asp-Pro peptide bond through a “twisted-amide” activation. Furthermore, in-frame deletion of the SPM domain from the ApxIVA protein of Actinobacillus pleuropneumoniae attenuated the virulence of this porcine pathogen in a pig respiratory challenge model. We hypothesize that the Ca^2+^-dependent clip-and-link activity represents an unconventional strategy for Gram-negative pathogens to adhere to the host target cell surface.

## Abstract (precis)

The Ca^2+^-dependent clip-and-link activity of large repeat-in-toxin (RTX) proteins is an exceptional posttranslational process in which an internal domain called a self-processing module (SPM) mediates Ca^2+^-dependent processing of a highly specific aspartate-proline (Asp-Pro) peptide bond and covalent linkage of the released aspartyl to an adjacent lysine residue through an isopeptide bond. Here, we report the solution structures of the Ca^2+^-loaded SPM (Ca-SPM) defining the mechanism of the autocatalytic cleavage of the Asp414-Pro415 peptide bond of the Neisseria meningitidis FrpC exoprotein. Moreover, deletion of the SPM domain in the ApxIVA protein, the FrpC homolog of Actinobacillus pleuropneumoniae, resulted in attenuation of virulence of the bacterium in a pig infection model, indicating that the Ca^2+^-dependent clip-and-link activity plays a role in the virulence of Gram-negative pathogens.

## INTRODUCTION

The Ca^2+^-dependent “clip-and-link” activity represents a distinct type of posttranslational autoprocessing of proteins. It consists of Ca^2+^-induced autocatalytic cleavage of a specific Asp-Pro peptide bond within a polypeptide precursor that generates a reactive C-terminal Asp anhydride, which can then react with an ε-amino group of an adjacent lysine residue to form a new covalent isopeptide bond (1). This process is mediated by a self-processing module (SPM), a 177-residue-long polypeptide domain that is exclusively present in a specific subset of repeat-in-toxin (RTX) proteins of Gram-negative bacteria (2). These very large proteins (>1,000 residues) are secreted by a dedicated type I secretion system (T1SS) that recognizes a C-terminal noncleavable secretion signal preceded by a variable number of tandem repeats of a consensus motif GGXGXDXXX (3). These RTX repeats form a calcium-binding parallel β-roll structure that folds vectorially, from the C to N terminus in the course of secretion, as the RTX substrates exit from the calcium-depleted bacterial cytosol into the calcium-rich extracellular milieu (4, 5). Even though many RTX proteins serve as major virulence factors of Gram-negative pathogens, the RTX proteins carrying the SPM domain were not found to exert any cytotoxic activity on host cells. Possibly, the Ca^2+^-dependent clip-and-link activity of FrpC, an iron-regulated RTX protein of Neisseria meningitidis, might be involved in the interaction of the bacterium with the host cell surface (6).

**FIG 1:**
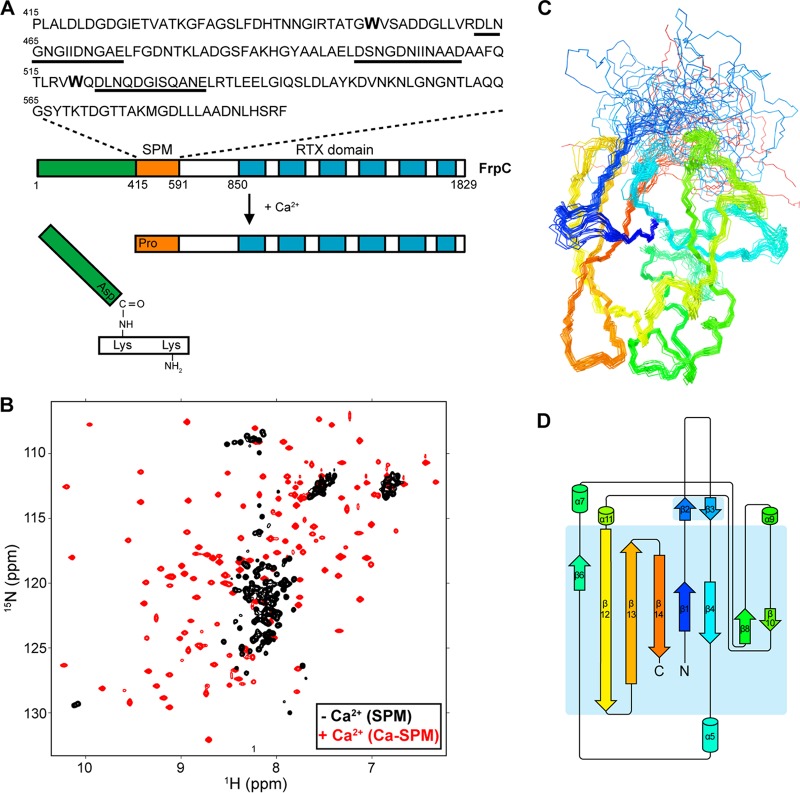
The NMR structure of the self-processing module (SPM) of the Neisseria meningitidis FrpC protein. (A) Schematic representation of the Ca^2+^-dependent clip-and-link of FrpC. The Ca^2+^-induced folding of FrpC is associated with a Ca^2+^-dependent conformational switch of SPM (residues 415 to 591 of FrpC, in orange), which promotes autocatalytic processing of the D_414_-P_415_ peptide bond and covalent linkage of the released D_414_ residue to an ε-amino group of a neighboring lysine residue through an Asp-Lys isopeptide bond. The residues of the putative EF-hand-like Ca^2+^-binding motifs are underlined. (B) Overlay of the ^1^H-^15^N HSQC spectra of ^15^N-labeled SPM in the absence (−) and in the presence (+) of 10 mM CaCl_2_. (C) Overlay of backbone traces of the 20 lowest energy structures of Ca-SPM solved by NMR, shown in a rainbow representation from blue (N terminus) to red (C terminus). (D) Topology of secondary structure elements of Ca-SPM generated by Pro-origami (55).

Recent biochemical and biophysical characterization of the SPM domain of FrpC revealed that the Ca^2+^-dependent clip-and-link activity results from the Ca^2+^-induced folding of the SPM polypeptide (2). Three EF-hand-like Ca^2+^-binding loops were predicted to be a key feature of an SPM structure, which appears to be stabilized by a noncovalent π-π interaction between two tryptophan residues (W_451_ and W_519_) arranged in a T-shaped orientation. Its formation involves a cooperative structural transition from an unfolded conformation to a well-folded state that enables a highly specific processing of the peptide bond between the Asp_414_ residue and the N-terminal Pro_415_ residue of the SPM amino acid sequence (residues 415 to 591 of FrpC). As a result, the D_414_-P_415_ bond breaks, and a reactive anhydride is formed at the newly released D_414_ residue, which upon nucleophilic attack by an ε-amino group of an adjacent polypeptide chain can form a new covalent isopeptide bond (Fig. 1A). Since the cross-linking activity of SPM can be inhibited by nucleophile scavengers, such as the reducing agents with a free thiol group (e.g., 1,4-dithiothreitol), the self-processing activity of SPM could be used for development of a self-excising affinity tag that allows purification of untagged recombinant proteins in a single chromatographic step (7). However, the structural and mechanistic aspects of the Ca^2+^-dependent clip-and-link activity of large RTX proteins remained unknown.

Here, we determined the solution structure of the calcium-loaded SPM (Ca-SPM) domain derived from the FrpC protein of Neisseria meningitidis and propose the mechanism of Ca^2+^-dependent autocatalytic processing of the D_414_-P_415_ peptide bond. We further show that abrogation of the Ca^2+^-dependent clip-and-link activity of ApxIVA, an exclusively *in vivo*-expressed RTX protein of Actinobacillus pleuropneumoniae, results in attenuation of the virulence of the pathogen in experimental lung infection in pigs. This is the first demonstration that the Ca^2+^-dependent clip-and-link activity of large RTX proteins plays a role in the virulence of Gram-negative pathogens.

## RESULTS

### NMR structure of Ca-SPM.

To gain insight into the catalytic mechanism of Asp-Pro bond processing, we determined the solution structure of calcium-loaded SPM (Ca-SPM) by nuclear magnetic resonance (NMR) spectroscopy. As shown in Fig. 1B, the ^1^H-^15^N heteronuclear single-quantum coherence (HSQC) spectrum of Ca-SPM exhibited the broad dispersion of backbone amide cross-peaks typical for structured proteins. The spectrum did not contain residual peaks typical for the ^1^H-^15^N HSQC spectrum of the Ca^2+^-depleted SPM (Fig. 1B, black contours), thus documenting that the Ca-SPM preparation was free of residual unfolded protein. Conventional triple resonance and nuclear Overhauser effect spectroscopy (NOESY) NMR spectra provided structural information for the whole protein, except of a region of 21 residues between T_430_ and G_450_. These residues were affected by conformational or chemical exchange and likely formed a loop lacking a unique structure. Moreover, deletion of the loop consisting of residues T_430_ to G_450_ did not affect the cleavage capacity of the respective glutathione *S*-transferase (GST)-SPM fusion protein, indicating that the loop does not contribute to structuring and autocatalytic activity of SPM (see Fig. S1 in the supplemental material). The overlay of 20 low-energy structures based on the 3,412 NOE and 234 dihedral angle restraints is displayed in Fig. 1C. The Ca-SPM structure has a compact fold composed of eight anti-parallel β-strands connected by a single helix-turn-helix motif and several surface-exposed turns and loops (Fig. 1D). These structures appear to be stabilized by calcium ions as the overall structure of Ca-SPM is well defined with a backbone root square mean deviation (RMSD) of 0.70 ± 0.16 Å (Table 1). However, the most abundant calcium isotope, ^42^Ca, is not observable by NMR, and the number and exact position of calcium ion(s) could not be identified by this NMR approach.

**TABLE 1:** NMR and refinement statistics for protein structures

Parameter	Value for the parameter in:			
	Ca-SPM	Ca-SPM-P415A	Ca-SPM +4 Ca^2+^ ions	SPM-P415A +5 Ca^2+^ ions
NMR distance and dihedral constraints				
Distance constraints (no.)				
Total NOE	3,412	2,250	3,412	2,250
Intraresidue	726	597	726	597
Interresidue	2,686	1,653	2,686	1,653
Sequential (|*i* – *j*| = 1)	853	552	853	552
Medium-range (|*i* – *j*| < 4)	460	293	460	293
Long-range (|*i* – *j*| > 5)	1,373	808	1,373	808
Total dihedral angle restraints (no.)				
Φ	117	119	117	119
ψ	117	119	117	119
Structure statistics				
Violations (mean ± SD)				
Distance constraints (Å)	0.03 ± 0.004	0.03 ± 0.007	0.03 ± 0.005	0.03 ± 0.006
Dihedral angle constraints (°)	0.40 ± 0.084	0.45 ± 0.099	0.50 ± 0.067	0.50 ± 0.097
Max dihedral angle violation (°)^*a*^	4.30	5.54	6.36	7.03
Max distance constraint violation (Å)	1.31	1.88	1.19	0.92
Deviation from idealized geometry^*b*^				
Bond lengths (Å)	1.14 ± 0.002	1.14 ± 0.002	1.14 ± 0.002	1.14 ± 0.002
Bond angles (°)	0.78 ± 0.01	0.79 ± 0.008	0.79 ± 0.007	0.79 ± 0.009
Impropers (°)	0.82 ± 0.03	0.83 ± 0.033	0.83 ± 0.027	0.83 ± 0.022
Avg pairwise RMSD (Å)^*c*^				
Heavy	0.95 ± 0.15	1.27 ± 0.21	0.94 ± 0.14	1.08 ± 0.16
Backbone	0.70 ± 0.18	0.97 ± 0.23	0.70 ± 0.16	0.80 ± 0.18

a Max, maximum.

b Calculated by WHAT IF.

c RMS Z-scores were calculated among 20 refined structures. The calculation includes the assigned residues 415 to 430 and 451 to 584.

10.1128/mBio.00226-20.1 FIG S1 The T430-G450 residues are not involved in the cleavage activity of SPM. Aliquots of the GST-SPM (left panel) and GST-SPMΔ430-450 (right panel) fusion proteins were incubated at the indicated concentrations of Ca^2+^ ions at 37°C for 30 min before being separated by 12% SDS-polyacrylamide gel electrophoresis. Download FIG S1, TIF file, 0.6 MB. Copyright © 2020 Kuban et al. 2020 Kuban et al. This content is distributed under the terms of the Creative Commons Attribution 4.0 International license.

### Calcium stoichiometry for Ca-SPM.

**FIG 2:**
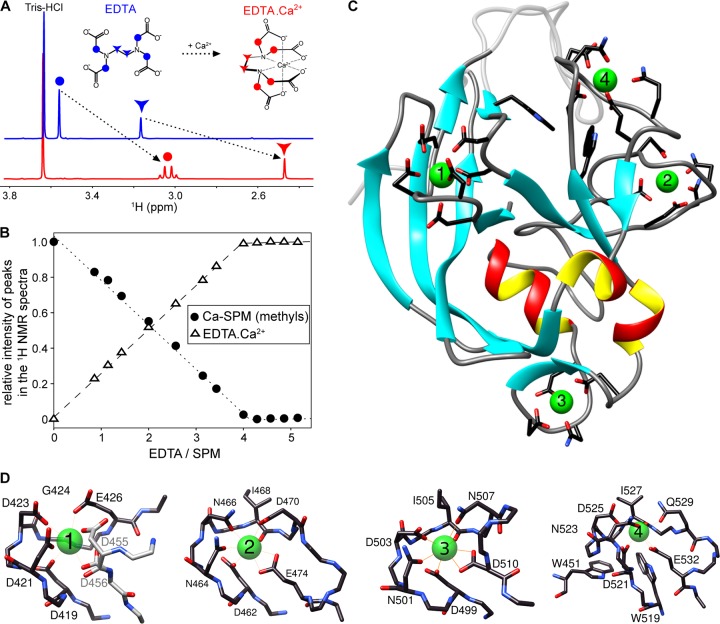
Ca-SPM binds four Ca^2+^ ions. (A) The ^1^H NMR spectra of free EDTA (blue) and the Ca^2+^-chelated EDTA (red). The positions of methylene and ethylene protons of the acetyl and ethylenediamine moieties are indicated by circles and triangles, respectively. (B) Titration of Ca-SPM by EDTA. Ca-SPM was dialyzed overnight at 4°C against the Ca^2+^-free buffer before being titrated by stepwise addition of EDTA. Relative intensities of methyl (circles) and Ca^2+^-chelated EDTA (triangles) peaks in the ^1^H NMR spectra were plotted against the molar EDTA/SPM ratio, with the maximal intensity of peak set arbitrarily to 1. (C) Structural model of the lowest energy structure of Ca-SPM. (D)Detailed view of the Ca^2+^-binding sites in Ca-SPM.

In order to determine the number of Ca^2+^-binding sites in SPM, Ca-SPM was dialyzed against a calcium-free buffer to remove free Ca^2+^ ions and was next subjected to NMR titration with the metal-chelating agent EDTA. The extensive dialysis of Ca-SPM did not affect the overall fold of the protein, indicating a high stability of the Ca-SPM structure at residual free Ca^2+^ concentrations. For precise quantification of bound Ca^2+^, we took advantage of the fact that complexation of free EDTA with Ca^2+^ ions is manifested by specific peak shifts in the ^1^H NMR spectra that are associated with changes in the chemical environment of methylene and ethylene protons of the acetyl and ethylenediamine moieties, respectively (8) (Fig. 2A). As demonstrated in Fig. S2, titration of Ca-SPM by EDTA resulted in progressive accumulation of singlet (2.47 ppm) and quartet (3.05 to 2.95 ppm) peaks in the ^1^H NMR spectra, corresponding to a gradual displacement of Ca^2+^ ions from Ca-SPM and formation of Ca^2+^-chelated EDTA (EDTA.Ca^2+^) complexes. This was further accompanied by a decrease of peak intensities in the aromatic, backbone amide and methyl regions of the ^1^H NMR spectra, illustrating an EDTA-induced unfolding of Ca-SPM. No peaks of partially folded SPM intermediates were detectable in the ^1^H-^15^N HSQC spectra, indicating a cooperative binding of Ca^2+^ ions (9). Once Ca-SPM was unfolded, additions of EDTA did not further affect the ^1^H NMR spectra, except of two singlet peaks at 3.5 and 3.15 ppm, which corresponded to proton signals from free EDTA (Fig. S2).

10.1128/mBio.00226-20.2 FIG S2 Overlay of one dimensional ^1^H NMR spectra over −1 to 11 ppm acquired during the stepwise titration of Ca-SPM by EDTA (upper panel). The unlabeled Ca-SPM protein (0.175 mM) in 0.54 ml of the Ca^2+^-free buffer (50 mM Tris-HCl, 50 mM NaCl, 10% D_2_O, 0.1% NaN_3_, and pH 7.4) was titrated with increasing concentrations of EDTA (50 mM stock solution), and the ^1^H NMR spectra of the SPM protein were successively recorded at indicated molar ratios (EDTA/protein). The backbone amide (left panel), EDTA and Tris (lower panel), and the methyl (right panel) regions of the ^1^H NMR spectra of the SPM protein are highlighted. The ^1^H frequencies of selected residues are indicated. Download FIG S2, TIF file, 1.0 MB. Copyright © 2020 Kuban et al. 2020 Kuban et al. This content is distributed under the terms of the Creative Commons Attribution 4.0 International license.

Changes in relative intensities of proton signals were used to determine Ca^2+^ stoichiometry for Ca-SPM. As shown in Fig. 2B, ∼4 molar equivalents of EDTA were required to completely unfold the Ca-SPM molecule. Moreover, the slope of the titration curve was close to −1/4, indicating a Ca^2+^ stoichiometry of 4:1 (Ca^2+^/SPM). In parallel, four calcium equivalents were required to saturate the signal of methylene protons of the Ca^2+^-EDTA complex, confirming that Ca-SPM contains four Ca^2+^-binding sites.

### Structural model of Ca-SPM.

The amino acid sequence of SPM has been predicted to contain three putative Ca^2+^-binding regions located between residues D_462_ and E_474_, D_499_ and D_511_, and D_521_ and E_532_ (2). These regions exhibit sequence homology to a 12-residue-long canonical EF-hand motif (D-X-[D/N]-X-[D/N]-G-X_5_-[D/E]) that binds the Ca^2+^ ion via side chain carboxyl (Glu or Asp) and carbonyl (Gln or Asn) groups in the conserved positions 1, 3, 5, and 12 (10). Visual inspection of the Ca-SPM structure revealed that the listed regions form very stable loops. In particular, the regions of D_499_ to D_511_ and of D_521_ to E_533_ form well-defined helix-loop-helix and helix-loop-strand structural motifs, respectively. Moreover, the SPM sequence contains a stretch of conserved residues (D_419_, D_421_, D_423_, and G_424_) that strongly resemble the conserved residues (positions 1, 3, 5, and 6) of an incomplete EF-hand-like motif. These residues form a stable loop in the Ca-SPM structure. Along with the adjacent D_456_ residue, these can provide two oxygens for calcium coordination and are likely to constitute the fourth Ca^2+^-binding site in Ca-SPM.

In order to build a reliable model of Ca-SPM, four Ca^2+^ ions were docked into four putative Ca^2+^-binding sites of the Ca-SPM structure and subjected to a series of molecular dynamics (MD) simulations. Analysis of the final Ca-SPM structures after 200 ns of unrestrained MD runs revealed that all four Ca^2+^ ions bind within the structure without triggering any significant conformational change at the level of the polypeptide backbone and without violating NMR restraints. However, calcium coordination provoked local structural changes at the level of side chains within Ca^2+^-binding loops, where the surface-exposed Asp or Glu residues were reoriented toward the Ca^2+^ ions. The reorientation and coordination of Ca^2+^ by the negatively charged residues was associated with a significant shift of the electrostatic surface potential to less negative values, except for the binding site of D_419_ to E_426_ that remained negative after Ca^2+^ binding (Fig. S3).

10.1128/mBio.00226-20.3 FIG S3 The electrostatic surface potential of the Ca-SPM protein in the absence (upper panel) and in the presence (lower panel) of four Ca^2+^ ions. The Ca^2+^-binding sites D419 to E426 and D456, D462 to E474, D499 to D510, and D521 to E532 are outlined by cyan, magenta, yellow, and green boundaries, respectively. The flexible regions (residues 430 to 450 and 585 to 593 containing four positively and four negatively charged residues) are not displayed. The potential ranges from −9 to 9 eV, with red and blue denoting negative and positive potential, respectively. Download FIG S3, TIF file, 1.3 MB. Copyright © 2020 Kuban et al. 2020 Kuban et al. This content is distributed under the terms of the Creative Commons Attribution 4.0 International license.

The final structural model of Ca-SPM is displayed in Fig. 2C. It is composed of eight anti-parallel β-strands, forming two β-sheets oriented perpendicularly to each other. The N-terminal β-strand is adjacent to the C-terminal β-strand (in antiparallel orientation), with the N and C termini of the molecule in close contact. Both β-strands form an interface between two β-sheets, constituting a hydrophobic core of the protein. This is enclosed by surface-exposed turns, a helix-turn-helix motif, and a long flexible loop at the N terminus of the protein. The structure is stabilized by binding of four Ca^2+^ ions, each coordinated by six oxygen atoms from side chains or backbone carbonyl groups of the engaged residues (Fig. 2D). Three of the four Ca^2+^-binding sites are made up of sequential motifs (D_462_ to E_474_, D_499_ to D_511_, and D_521_ to E_532_) that closely resemble that of EF hands, while the fourth Ca^2+^-binding site consists of a structural motif, where the position 12 of an incomplete EF-hand-like motif (D_419_ to G_424_) is structurally supplemented by D_456_. The calcium-binding site at D_521_ to E_532_ is adjacent to a specific pair of two tryptophan residues (W_451_ and W_519_), whose aromatic rings are arranged in the T-shaped or edge-to-face orientation (center-to-center distance of 6.1 ± 0.3 Å; angle between the ring planes of 85 ± 5°).

### Ca-SPM adopts a novel fold.

Structural similarity searches using CATH (11), COFACTOR (12), DALI (13), and PDBeFold (14) revealed that the Ca-SPM structure does not resemble any of the structures deposited in the Protein Data Bank (PDB) (Fig. S4). No significant hits were found using DALI and COFACTOR (template-modeling [TM] score of <0.5 and Z score of <2). The closest structural match was identified between the Ca-SPM structure and the human split pleckstrin homology domain of phospholipase C-γ (PDB 2W2W). However, the overall homology was very low, with a Q-score of 0.12. Hierarchic classification of protein domain structures using CATH revealed that the Ca-SPM structure shows signs of similarity with a β-barrel of the lipocalin fold (CATH superfamily 2.40.128.50) of d-aminopeptidase from the Gram-negative bacterium Ochrobactrum anthropi and a two-layer sandwich domain (CATH superfamily 3.30.60.30) of a wild turkey serine protease inhibitor. However, the CATH analysis yielded SSAP (sequential structure alignment program) scores of 71.6 and 72.1 (with RMSDs of 5.1 Å and 9.8 Å for 82 and 51 residues, respectively), indicating that the Ca-SPM structure does not contain the same fold and only belongs to the same protein class with common structural motifs. Hence, the structure of Ca-SPM reveals a novel fold.

10.1128/mBio.00226-20.4 FIG S4 Overlay of the Ca-SPM structure (brown) with (a) the human split pleckstrin homology domain of phospholipase C-γ (light blue; PDB entry code 2W2W), (b) Ochrobactrum anthropi d-aminopeptidase (dark blue; PDB entry code 1EI5), and (c) serine protease inhibitor (cyan; PDB entry code 1R0R) from Bacillus licheniformis. Download FIG S4, TIF file, 2.4 MB. Copyright © 2020 Kuban et al. 2020 Kuban et al. This content is distributed under the terms of the Creative Commons Attribution 4.0 International license.

### Backbone dynamics of Ca-SPM.

In order to assess the dynamic properties of Ca-SPM on a fast (subnanosecond) timescale, we measured the steady-state ^1^H-^15^N nuclear Overhauser enhancement and the longitudinal (*R*_1_) and transverse (*R*_2_) relaxation rates (Fig. S5). Based on a model-free approach, the analysis yielded the overall rotational correlation time (τ_c_) of 7.54 ± 0.01 ns. The generalized order parameter *S*^2^, describing the amplitude of fast (picosecond) internal motions, was higher than 0.8, indicating that the Ca-SPM backbone is rigid, similar to that of other well-folded proteins. Lower *S*^2^ values (<0.8) were observed only for six residues (G_422_, L_463_, D_484_, S_500_, V_551_, and Q_563_), illustrating high flexibility of these residues within the Ca^2+^-binding sites. Moreover, the NMR relaxation experiments revealed a conformational exchange on the micro- to millisecond timescale: (i) the *R*_2_ values were elevated (>15 s^−1^) for residues V_428_ and I_468_ to A_493_, and (ii) the ratio between the intensities of the ^1^H-^15^N cross-peaks at 30°C and 10°C (*I*_30°C_/*I*_10°C_) was decreased for residues in the regions of I_425_ to L_456_, I_468_ to D_493_, and Q_520_ to R_534_ against the expected *I*_30°C_/*I*_10°C_ value. Most of these residues are in close proximity to the invisible portion of the structure, documenting that the region of T_430_ to G_450_ is in a slow conformational exchange.

10.1128/mBio.00226-20.5 FIG S5 NMR protein dynamics characterization of Ca-SPM. The backbone amide ^15^N *R*_1_ longitudinal (a) and *R*_2_ transverse (b) relaxation rate parameters and the steady-state ^15^N-^1^H NOE (c) were measured at 30°C, and the values were plotted against the residue number. In panel b, the experimental data are shown in black dots, and the calculated chemical (conformational) exchange contribution to *R*_2_ (*R*_ex_) is depicted as red columns. The apparent order parameter (*S*^2^) was derived from the model-free approach and is plotted in panel d. (e) Ratio of peak intensities in the ^1^H-^15^N HSQC spectra acquired at 30°C and 10°C (*I*_30°_/*I*_10°_). The peak intensity ratio of 1.75 (grey line) represents an expected ratio caused only by a change of water viscosity. The values below the limit represent a conformational (or chemical) exchange resulting in line broadening at the higher temperature. Download FIG S5, TIF file, 0.9 MB. Copyright © 2020 Kuban et al. 2020 Kuban et al. This content is distributed under the terms of the Creative Commons Attribution 4.0 International license.

### Solution structure of a catalytic intermediate of SPM (SPM-P415A).

**FIG 3:**
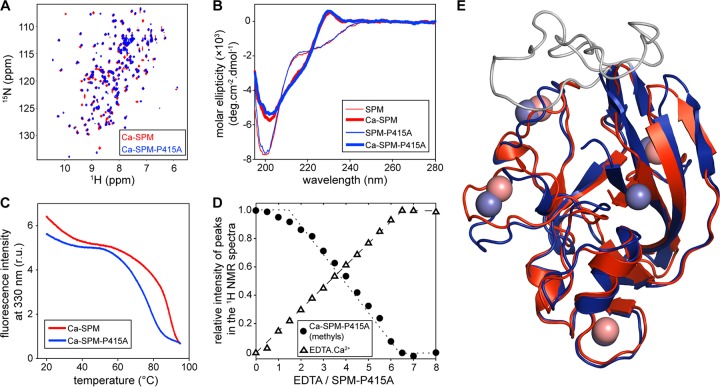
The NMR structure of the cleavage-incompetent mutant Ca-SPM-P415A. (A) Overlay of the ^1^H-^15^N HSQC spectra of ^15^N-labeled Ca-SPM and Ca-SPM-P415A, as indicated. (B) Circular dichroism spectra of SPM and SPM-P415A in the absence (thin line) or presence (thick line) of 10 mM CaCl_2_. (C) Thermal unfolding of Ca-SPM and Ca-SPM-P415A in the presence of 10 mM CaCl_2_ as assessed by nano-differential scanning fluorimetry. (D) Titration of Ca-SPM-P415A by EDTA. Ca-SPM-P415A was dialyzed overnight at 4°C against the Ca^2+^-free buffer before 2 molar Ca^2+^ equivalents were added to complete the folding of Ca-SPM-P415A. The sample was titrated by stepwise addition of EDTA, and relative intensities of methyl and Ca^2+^-chelated EDTA (EDTA.Ca^2+^; triangles) peaks in the ^1^H NMR spectra were plotted against the molar EDTA/SPM-P415A ratio. (E) Overlay of ribbon representation of the NMR structure of Ca-SPM (red) and Ca-SPM-P415A (blue).

To unravel the mechanistic details of the D_414_-P_415_ cleavage, we prepared a cleavage-incompetent mutant of SPM, where the proline residue (P_415_) of the D_414_-P_415_ cleavage site was replaced by an alanine residue (P415A). Unlike Ca-SPM, which represents a stabilized low-energy conformation of SPM after the cleavage and in which the N-terminal sequence begins with the P_415_ residue (_415_PLALD_419_), the SPM-P415A construct possesses the GSDALALD_419_ sequence at its N terminus. This is not processed, and it is likely to represent a structure of the SPM precursor before cleavage. As shown in Fig. 3A, the overlay of the HSQC spectra demonstrated that the chemical shift patterns of the Ca^2+^-saturated SPM-P415A (Ca-SPM-P415A) and Ca-SPM almost overlap, indicating that the cleavage of the D_414_-P_415_ peptide bond is preceded by Ca^2+^-induced folding of the SPM polypeptide and that the overall structure of SPM remains unaffected after the cleavage.

Calcium titration followed by far-UV circular dichroism (CD) spectroscopy revealed that the SPM-P415A protein undergoes Ca^2+^-induced structural transition from an unfolded to a folded conformation that is undistinguishable from that of SPM (Fig. 3B). Moreover, Ca-SPM-P415A exhibited a strong CD exciton couplet at 230 nm, indicating that the T-shaped orientation of two tryptophan residues was maintained in the Ca-SPM-P415A structure. However, thermal unfolding experiments performed by nano-differential scanning fluorimetry (nanoDSF) revealed that Ca-SPM-P415A unfolds at a lower temperature than Ca-SPM, with the midpoint of the thermal melting curves (or melting temperature, *T_m_*) being shifted by about 10°C (from 89°C to 79°C) (Fig. 3C). The *T_m_* values were dependent on Ca^2+^ concentrations in the sample buffer, and reversible unfolding of the proteins enabled us to determine thermodynamic parameters of the folding/unfolding process. The values of Gibbs free energy of Ca-SPM and Ca-SPM-P415A unfolding, calculated for the Ca^2+^-saturated protein (10 mM CaCl_2_) at 30°C, were determined to be approximately 80 kJ/mol and 40 kJ/mol, respectively. Thus, the three extra residues at the N terminus negatively affected the thermal stability of Ca-SPM-P415A, indicating structural destabilization of the overall structure prior to cleavage.

The number of Ca^2+^-binding sites in Ca-SPM-P415A was determined by NMR titration experiments. Unlike results for Ca-SPM, the dialysis of Ca-SPM-P415A against the Ca^2+^-free buffer yielded a partial unfolding of the protein, indicating a lower conformational stability of the Ca-SPM-P415A structure. In order to avoid inaccurate interpretation of the titration data, the dialyzed Ca-SPM-P415A sample was supplemented with an excess of two molar equivalents of Ca^2+^ (to ensure folding of 100% molecules) and was next titrated with EDTA. As shown in Fig. 3D, the EDTA-induced unfolding of Ca-SPM-P415A was characterized by a nonlinear equilibrium and was completed after addition of ∼6.5 equivalents of EDTA (including two Ca^2+^ equivalents added prior to the EDTA titration). The titration data fit well to binding models describing the cooperative binding of both four and five Ca^2+^ ions, but none of the models could unambiguously assign the exact number of Ca^2+^-binding sites in the Ca-SPM-P415 structure. However, the overall calcium stoichiometry for Ca-SPM-P415A was higher than that for Ca-SPM (Fig. 2B), indicating the presence of an additional Ca^2+^-binding site in the Ca-SPM-P415A structure.

The solution structure of Ca-SPM-P415A was determined using a procedure identical to that for Ca-SPM. A total of 2,250 distance and 119 dihedral angle restraints were used for the structure calculations (Table 1), and the overlay of final structures is depicted in Fig. 3E. Comparison of the Ca-SPM-P415A structure with that of Ca-SPM revealed that the two structures are highly similar across the models, with the average RMSD of 1.12 Å for the assigned residues. Differences in chemical shifts in most of the residues were very low (<0.1 ppm), except for residues located at the N and C termini (L_416_ and G_578_ to L_582_) and the loop adjacent to the N terminus (G_477_ to N_479_ and H_490_). Close examination of the Ca-SPM-P415A structure revealed that the N terminus of the protein adjoins two carboxy side chains of D_478_ and D_579_, which were proposed to be involved in the catalysis of the D_414_-P_415_ peptide bond cleavage by promoting protonation of P_415_ (1). Moreover, D_478_ and D_579_ along with D_414_ form a region with a negative net charge in the vicinity of the cleavage site, which is likely to form the fifth Ca^2+^-binding site in the Ca-SPM-P415A structure. Therefore, the fifth Ca^2+^ ion, coordinated by D_414_, D_478_, and D_579_, was modeled within the Ca-SPM-P415A structure.

### D_478_ and D_579_ residues do not catalyze the D_414_-P_415_ peptide bond cleavage.

To assess whether the carboxylate anions of D_478_ and D_579_ residues catalyze the D_414_-P_415_ peptide bond cleavage, the D_478_ and D_579_ residues were replaced by alanine or asparagine residues, and the Ca^2+^-dependent self-processing activity of the mutants was examined using the respective GST-SPM fusion proteins. As shown in Fig. S6, the replacement of D_478_ with alanine (D478A) resulted in a significant reduction of the self-processing activity, while only a moderate reduction in the cleavage rate was observed upon replacement of D_478_ by asparagine (D478N). In contrast, the removal of the carboxyl group from D_579_ completely abolished the Ca^2+^-dependent processing of the GST-SPM-D579A protein, while the D579N substitution had no significant impact on the cleavage of the GST-SPM-D579N protein. The double D478N D579N substitution reduced but did not eliminate the cleavage activity of SPM, indicating that the carboxyl groups of the D_478_ and D_579_ residues do not catalyze the cleavage of the D_414_-P_415_ peptide bond but, rather, play a key structural role.

10.1128/mBio.00226-20.6 FIG S6 D478 and D579 residues do not catalyze the Asp-Pro peptide bond cleavage. Aliquots of the indicated GST-SPM fusion proteins were incubated at the indicated concentrations of Ca^2+^ ions at 37°C for 30 min before being separated by 12% SDS-polyacrylamide gel electrophoresis. Download FIG S6, TIF file, 1.6 MB. Copyright © 2020 Kuban et al. 2020 Kuban et al. This content is distributed under the terms of the Creative Commons Attribution 4.0 International license.

### Structural destabilization of the SPM precursor in simulations.

**FIG 4:**
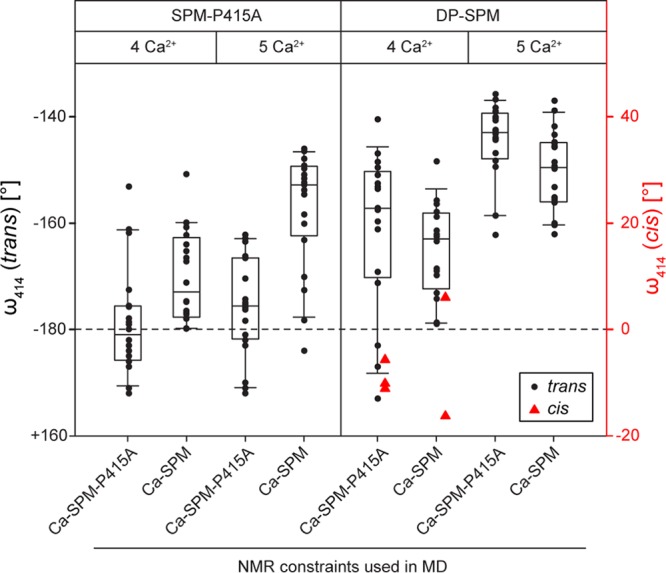
Calcium coordination by D_414_, D_478_, and D_579_ augments the distortion of the scissile D_414_-P_415_ peptide bond. A total of four or five calcium ions were docked into the Ca^2+^-binding sites of the Ca-SPM-P415A structure or the structural model of an SPM precursor (originating from replacement of A_415_ by a proline residue in the Ca-SPM-P415A structure to restore the D_414_-P_415_ cleavage site [DP-SPM]) and subjected to molecular dynamics (MD) simulations consisting of simulated annealing and cooling in the presence of the experimental NMR restraints (Ca-SPM or Ca-SPM-P415). The degree of rotation of the D_414_-P_415_ peptide bond is expressed as the deviation of the ω_414_ dihedral angle from its near planar geometry (ω_414_ = 180°, dashed line). The box plots represent the median values, with upper and lower quartiles obtained from 20 calculations. Structures adopting the *cis* conformation (ω_414_ = 0°) of the D_414_-P_415_ peptide bond are indicated.

To get insight into structure of the autoproteolytic site prior to cleavage, the A_415_ residue in the Ca-SPM-P415A structure was replaced *in silico* by a proline, and the resulting structural model of an SPM precursor (DP-SPM) was subjected to molecular dynamics (MD) simulations. The purpose of the analysis was not to determine the exact value of the ω_414_ dihedral angle (which is usually planar and fixed to 180°) but to monitor the balance between violation of an ideal peptide bond geometry (strongly imposed by the force field regardless of the actual chemical context) and the overall structure derived from the experimental data and force field requirements. The MD simulations revealed that the noncleavable D_414_-A_415_ peptide bond in the Ca-SPM-P415A structure containing four Ca^2+^ ions is almost completely in *trans* conformation (ω_414_ = 180°). This is in line with the experimental data and validates our computational approach (Fig. 4). In contrast, the Ca-SPM-P415A structure containing five Ca^2+^-binding sites exhibited a significant distortion of the D_414_-A_415_ peptide bond geometry, with a deviation from a near planar geometry by about 25° and 15° (depending on the NMR experimental restraints used in the calculations). In line with that, the D_414_-P_415_ peptide bond in the DP-SPM structure containing four Ca^2+^ ions had a deviation of about 20° and even 40° in a structural model containing five Ca^2+^ ions. The *cis* isomer of ω_414_ occasionally appeared during simulations but with experimental restraints violated (Fig. 4). Taken together, these data suggested that the autocatalytic cleavage of the D_414_-P_415_ peptide bond is associated with a ground-state destabilization of the D_414_-P_415_ peptide bond through a twisted amide.

### The Ca^2+^-dependent clip-and-link activity of the ApxIVA protein plays an important role in A. pleuropneumoniae infection.

**FIG 5:**
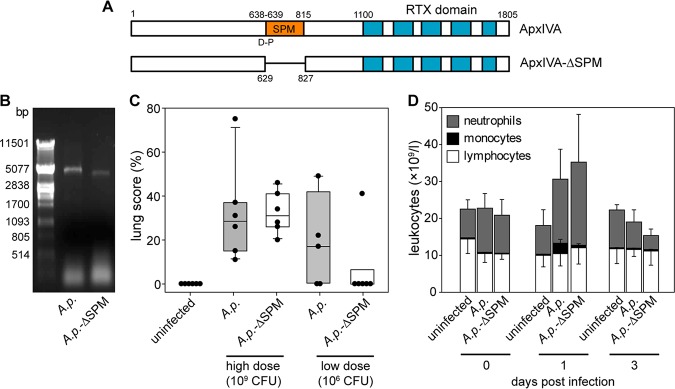
The Ca^2+^-dependent clip-and-link activity of ApxIVA plays an important role in Actinobacillus pleuropneumoniae infection. (A) Schematic representation of the ApxIVA constructs used in this study. The ApxIVA processing site is located between the D_638_ and P_639_ residues, followed by the SPM residues (639 to 815). (B) The PCR amplification of the *apxIVA* gene from the genomic sequence of the wild-type and the ΔSPM strains of A. pleuropneumoniae. (C) Gross lung pathology of pigs challenged with A. pleuropneumoniae. Groups of six animals each were uninfected or intranasally infected with the wild-type and the ΔSPM strains of A. pleuropneumoniae at high (1 × 10^9^ CFU per animal) and low (1 × 10^6^ CFU per animal) doses. The total lung scores were determined at days 1 and 3 postinfection in animals infected with the high and low bacterial doses, respectively. (D) Differential leukocyte counts in the peripheral blood of pigs challenged with the low dose of A. pleuropneumoniae cells. *A.p*., A. pleuropneumoniae.

Since N. meningitidis is an exclusively human pathogen, the existing animal models do not accurately simulate meningococcal disease. It has thus proved difficult to study the role of individual proteins involved in the pathogenesis of N. meningitidis *in vivo*. However, the Ca^2+^-dependent clip-and-link activity has been observed also for ApxIVA (1), one of the RTX proteins of the animal pathogen A. pleuropneumoniae that causes porcine pleuropneumonia infections (15). Like FrpC, ApxIVA comprises a highly conserved SPM segment (residues 639 to 815 of the full-length ApxIVA) that mediates Ca^2+^-dependent cleavage of the D_638_-P_639_ peptide bond and a covalent linkage of the processed N-terminal segment to adjacent proteins (1) (Fig. 5A). To assess the biological importance of the Ca^2+^-dependent clip-and-link activity of ApxIVA *in vivo*, we introduced an in-frame deletion of codons 629 to 827 in the *apxIVA* gene on A. pleuropneumoniae chromosome (Fig. 5B) and examined the virulence of the mutant strain (ΔSPM) in a pig infection model.

Intranasal administration of high doses (∼10^9^ CFU per animal) of the wild-type and mutant strains provoked an acute form of pleuropneumonia, causing deaths of most of piglets within 24 h. The surviving animals were in the terminal stage of the disease and were euthanized at day 1 postinfection. Postmortem examination revealed that the lungs of infected animals exhibited the characteristic, dark red-to-black necro-hemorrhagic areas of consolidation accompanied by fibrinous pleuritis, with an average lung score of 28 and 32% for the wild-type and the mutant strains, respectively (Fig. 5C). However, at lower bacterial challenge doses (∼10^6^ CFU per animal), moderate clinical symptoms were elicited, including lethargy, respiratory distress, and tachypnea, with no pleuropneumonia-related deaths during the time frame of the experiment (3 days). The body (rectal) temperature was largely above the physiological range (>40.5°C) and remained elevated for about 48 h. Regular hematological examinations showed neutrophilic leukocytosis, reaching the maximum at day 1 postinfection in all infected animals (Fig. 5D). However, compared to the challenge with the wild-type bacteria, no monocytosis was observed in pigs challenged with the ΔSPM mutant, and the overall monocyte counts were the same as for uninfected controls. Moreover, the average lung score for the ΔSPM-challenged pigs was lower than for pigs challenged with the wild-type strain (6.8 versus 14.7%), although the wild-type challenge strain was reisolated from lungs of only 3 out of 6 animals (compared to 5 of 6 animals for the ΔSPM strain). This result showed a significant reduction of lung lesions in pigs challenged with the ΔSPM strain, suggesting that the Ca^2+^-dependent clip-and-link activity of ApxIVA plays a specific role in the pathogenesis of porcine pleuropneumonia.

## DISCUSSION

Ca^2+^-dependent protein clip-and-link activity of large RTX proteins represents a unique mechanism of posttranslational processing of proteins that involves a rearrangement of a polypeptide backbone through the highly specific Ca^2+^-dependent autocatalytic cleavage of an Asp-Pro peptide bond and a nonspecific covalent linkage of the released carboxyl of the Asp residue to ε-amino groups of adjacent Lys residues. The cleavage of the Asp-Pro peptide bond is catalyzed by the Ca^2+^-dependent structural transition of the SPM domain, which is highly conserved and defines a specific subclass of RTX exoproteins (1, 2). Very low Ca^2+^ concentrations in the bacterial cytosol (<100 nM) maintain the RTX proteins in an unfolded state that is required for single-step translocation across the bacterial envelope via the T1SS apparatus (16). Once exported to the calcium-rich extracellular environment (>100 μM), the proteins fold and acquire their biological activity. Given the fact that the binding affinity of SPM for Ca^2+^ is about 150 μM (2), the Ca^2+^-dependent clip-and-link activity of the RTX proteins occurs exclusively outside the bacteria.

In this work, we determined the solution structure of SPM of the N. meningitidis FrpC protein and provided the structural insight into the Ca^2+^-dependent cleavage of the D_414_-P_415_ peptide bond. The Asp-Pro bond belongs to the most labile peptide bond, which is known to be selectively hydrolyzed within several days under acidic conditions at higher temperatures (17–20). This is due to a higher basicity of the secondary amine group in the proline than that of the primary amine group of other amino acids. At low pH, the free electron pair on the proline nitrogen may polarize the carbonyl C-N bond and make the carbonyl carbon more susceptible to nucleophilic attack. The increased basicity of the nitrogen atom may promote protonation of the nitrogen atom of the intermediate, resulting in the cleavage of the Asp-Pro peptide bond. In contrast, the SPM-mediated cleavage of the Asp-Pro peptide bond is a highly specific catalytic reaction that occurs quickly at physiological pH and with reaction rates of about 2 orders of magnitude higher than the uncatalyzed chemical cleavage. Osicka and coworkers (1) proposed that the protonation of the proline nitrogen in SPM could be promoted by an as yet uncharacterized residue(s), which can interact with the proline residue. Inspection of the Ca-SPM-P415A structure revealed two aspartate residues (D_478_ and D_579_) positioned in close proximity to P_415_. However, site-directed mutagenesis ruled out that any of the aspartates acted as the proton-donating amino acid (see Fig. S6 in the supplemental material). Instead, both aspartates appear to play a structural role in binding of an additional (fifth) calcium ion. The fifth calcium-binding site was deduced to reside only in the SPM-P415A structure, indicating the essential structural role of the D_414_ residue prior to cleavage. Molecular docking revealed that the fifth calcium ion could be coordinated by the carbonyl oxygen of the D_414_ residue and together with D_478_ and D_579_ would constitute a transient calcium-binding site that is formed during the Ca^2+^-dependent folding of SPM and falls apart upon the cleavage of the D_414_-P_415_ peptide bond. The binding of the fifth calcium ion enhances distortion of the C-N bond of the carboxyamide group, generating a twisted amide in a scissile bond, which destabilizes the overall structure of SPM. This is well documented by the experimental data showing that the thermal stability of the Ca-SPM-P415A structure before cleavage is much lower than that of the Ca-SPM structure after the cleavage.

**FIG 6:**
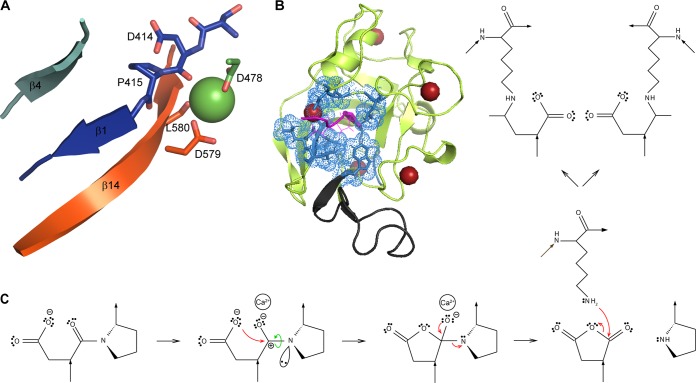
Structure-functional model of the Ca^2+^-dependent clip-and-link activity of N. meningitidis FrpC. (A) A detailed view into the putative structure of the D_414_-P_415_ cleavage site of SPM before the autocatalytic cleavage. The N-terminal segment of SPM is structurally constrained by bending of the D_414_-P_415_ peptide bond over the C-terminal β14-strand in an SPM precursor structure, which results in deviation of the scissile bond from its nearly planar geometry. Coordination of calcium ion by the carbonyl groups of D_414_ and L_580_ along with the carboxy groups of the D_478_ and D_579_ residues augments the rotation of the D_414_-P_415_ peptide bond to generate a twisted amide. A cartoon representation of SPM is shown in rainbow colors from the N terminus in blue to the C terminus in red. The calcium ion is represented by green ball. (B) The D_414_-P_415_ cleavage site is enclosed by a narrow port. The residues of the narrow port (W_451_, L_475_, G_477_, G_491_, and D_579_ to L_582_) are highlighted in blue. (C) A proposed chemistry of the Ca^2+^-dependent autocatalytic processing of the D_414_-P_415_ peptide bond and formation of a new Asp_414_-Lys isopeptide bond. The Ca^2+^-dependent assembly of the SPM precursor is associated with structural constraint of the D_414_-P_415_ linkage that promotes rotation of the scissile bond to generate a twisted amide (green arrow). The formation of the twisted amide is further facilitated by the presence of a calcium ion coordinated by the carbonyl group of D_414_ and two carboxy groups provided by D_478_ and D_579_. The twisted amide generates a reactive lone electron pair at the nitrogen that abolishes the conjugation of the nitrogen with the carbonyl π electrons, enabling a nucleophilic attack of the carboxy group of D_414_ on its carbonyl carbon. This results in formation of a cyclic imide intermediate and rupture of the imide bond to P_415_ with subsequent formation of a cyclic D_414_ anhydride. The reactive anhydride at D_414_ can be attacked at carboxy carbons of the anhydride ring by the free amino group of a lysine residue, leading to formation of an isopeptide bond with either α- or β-carboxyl of the D_414_ residue.

Twisted amides represent one of the approaches for activation of amide bonds, which are usually stable due to formation of a resonating structure provided by the conjugation of the nitrogen lone pair with the carbonyl group (21, 22). The distortion of amide bonds causes a loss of double-bond character and thus increases their reactivity compared to planar counterparts. Such an unusual conformation of the scissile amide bond is the driving force for a nucleophilic or electrophilic attack that is proposed to be a central design element of a variety of enzymatic processes, such as *cis*-*trans* isomerization (23), amide hydrolysis (24), N-linked glycosylation of proteins (25), and intein-based protein splicing (26). Based on the data above, we propose a mechanism for the SPM-mediated processing of the Asp-Pro peptide bond (Fig. 6). Upon calcium binding, the SPM polypeptide undergoes a highly cooperative structural transition from an intrinsically unstructured conformation to the compact protein fold that is stabilized by binding of four calcium ions in EF-hand-like Ca^2+^-binding sites. The scissile D_414_-P_415_ peptide bond adopts a highly strained turn that is structurally constrained by the adjacent β1-strand and the residues of a narrow port (W_451_, L_475_, G_477_, G_491_, and D_579_ to L_582_) that shields the D_414_-P_415_ cleavage site from the solvent (Fig. 6A and B). Coordination of the fifth calcium ion by the oxygen atom of the carbonyl group of D_414_ provokes rotation of the D_414_-P_415_ peptide bond over the C-terminal β14-strand that apparently accentuates rotation of the C-N bond to produce a twisted amide. Such a distortion generates a reactive lone electron pair at the nitrogen that abolishes the conjugation of the nitrogen electrons with the carbonyl group and facilitates a nucleophilic attack of the carboxylic acid side chain of D_414_ on its carbonyl carbon (Fig. 6C). This results in formation of a cyclic imide intermediate and D_414_-P_415_ peptide bond cleavage, with subsequent formation of a reactive cyclic D_414_ anhydride. This can next be attacked by a free amino group of a lysine residue to generate an isopeptide bond.

The SPM represents a highly conserved domain that appears to be present in many RTX proteins of Gram-negative bacteria. However, the SPM-mediated processing of the Asp-Pro peptide bond has as yet been reported only for the N. meningitidis FrpC and A. pleuropneumoniae ApxIVA proteins *in vitro* (1). Sviridova et al. (6) demonstrated that incubation of meningococci with human alveolar A549 cells resulted in the processing of the D_414_-P_415_ peptide bond of the freshly secreted FrpC and a covalent linkage of the FrpC_1–414_ segment to cell surface proteins of the epithelial cells. In view of the fact that FrpC_1–414_ exhibits a very high affinity (dissociation constant [*K_d_*] of ∼0.2 nM) for FrpD, the meningococcal outer membrane lipoprotein encoded in the same operon upstream of the *frpC* gene (27), the interaction of FrpC_1–414_ with FrpD could be involved in tight adherence of N. meningitidis to the host cell surface. Nevertheless, deletion of the *frpC* gene from the genome of N. meningitidis MC58 did not affect the mortality rate of rats after a systemic meningococcal infection, indicating that FrpC is dispensable for virulence in the infant rat model (28). However, this does not preclude the role of the SPM-mediated processing of FrpC in the attachment of meningococci to mucosal surfaces that are the colonization niche of N. meningitidis.

Our *in vivo* pig experiments showed that the abrogation of the Ca^2+^-dependent clip-and-link activity of ApxIVA led to significant reduction of necrotizing lung lesions after A. pleuropneumoniae infection (Fig. 5C). These data are in good agreement with the previous result showing that the deletion of the *apxIVA* gene attenuates the virulence of A. pleuropneumoniae in a pig infection model (29). A. pleuropneumoniae produces four different RTX proteins, where only ApxI, ApxII, and ApxIII are toxins possessing both hemolytic and cytotoxic activity (30). With respect to the fact that the fourth RTX protein, ApxIVA, does not exert any of these activities, the biological activity of ApxIVA could be ascribed to the Ca^2+^-dependent clip-and-link activity. It is tempting to speculate that colonization of pig respiratory airways by A. pleuropneumoniae is associated with the Ca^2+^-dependent processing of the D_638_-P_639_ peptide bond of the type I-secreted ApxIVA and covalent linkage of the ApxIVA_1–638_ fragment to the host respiratory epithelium, which would serve as a high-affinity target for an as yet unidentified protein structure exposed on the bacterial cell surface. This would allow tight adherence of the bacterial cells to the host respiratory epithelia, which may be essential for the full virulence of A. pleuropneumoniae. Hence, covalent attachment of the N-terminal fragments of specific RTX proteins to the host proteins through the Ca^2+^-dependent protein clip-and-link activity could represent an unconventional strategy for pathogenic microorganisms to adhere to the target host cell surface.

## MATERIALS AND METHODS

### Reagents.

The suicide vector for allelic exchange on the Actinobacillus pleuropneumoniae chromosome (pEMOC2) and the Escherichia coli β2155 (Δ*dapA*) strain were generously provided by Gerald F. Gerlach (Institute for Innovative Veterinary Diagnostics, Hannover, Germany).

### Plasmid construction.

The wild-type SPM protein was expressed from the pET28b-dHis-GST-SPM plasmid as a C-terminal glutathione *S*-transferase (GST) fusion protein as previously described (31). The SPM-P415A protein was expressed from the pET42b-SPM-P415A plasmid generated by PCR amplification of the nucleotide sequence of SPM from the pET28b-dHis-GST-SPM construct using a pair of forward and reverse primers (5′-TTTACTAGTGAAAACCTGTACTTCCAGGGCAGCGCTCCGCTAGCCC-3′ and 5′-TTTCCATGGTTACTCGAGGAAGCGGCT-3′) and ligation of the SpeI/NcoI-restricted PCR fragment into the SpeI/NcoI-cleaved pET42b vector. The pET28b-dHis-GST-SPM_Δ430–450_ construct was prepared by PCR mutagenesis using a pair of primers (5′-GGGTCGGGATCCGAATTC-3′ and 5′-AAAACCGGTAACGGTTTCTATGCCGTCG-3′) and subcloning of the BamHI*/*AgeI-restricted PCR fragment into the BamHI*/*AgeI-cleaved pET28b-dHis-GST-SPM plasmid. The pET28b-dHis-GST-SPM-D579A construct was prepared by PCR mutagenesis, whereby two PCR fragments each carrying the linking ApaI restriction site were amplified by PCR using two pairs of PCR primers (5′-GCCACCGGTTGGGTTTC-3′ and 5′-TTTGGGCCCCCATTTTTGCGGTTG-3′; 5′-TTTGGGCCCTGCTTTTAGCAGCCGACAAC-3′ and 5′-GGCCCACTACGTGAACC-3′), cleaved with the respective pair of restriction enzymes (ApaI*/*AgeI and ApaI/DraIII), and collectively ligated into the AgeI/DraIII-cleaved pET28b-dHis-GST-SPM plasmid. The pET28b-dHis-GST-SPM-D579N plasmid was constructed under the same conditions except that the PCR primer pair 5′-GCCACCGGTTGGGTTTC-3′ and 5′-TTTAAATTCCCCATTTTTGCGGTTGTAC-3′ and the pair 5′-TTTGAATTTACTTTTAGCAGCCGACAAC-3′ and 5′-GGCCCACTACGTGAACC-3′ and the ApoI restriction site were used. The pET28b-dHis-GST-SPM-D478A construct was prepared by site-directed overlap PCR mutagenesis using two pairs of PCR primers (5′-GACAAGCTTCATATGTCCC-3′ and 5′-CTGCCAGTTTGGTATTCGCGCCGAAGAGTTCCGCGC-3; 5′-GCGCGGAACTCTTCGGCGCGAATACCAAACTGGCAG-3′ and 5′-GGCCCACTACGTGAACC-3′), whereby the resulting HindIII/DraIII-restricted PCR product was cloned into the HindIII/DraIII-cleaved pET28b-dHis-GST-SPM plasmid. The pET28b-dHis-GST-SPM-D478N plasmid was constructed under the same conditions except that the PCR primer pair 5′-GACAAGCTTCATATGTCCC-3′ and 5′-CTGCCAGTTTGGTATTGTTGCCGAAGAGTTCCGCGC-3′ and the pair 5′-GCGCGGAACTCTTCGGCAACAATACCAAACTGGCAG-3′ and 5′-GGCCCACTACGTGAACC-3′ were used. The double-substitution construct pET28b-dHis-GST-SPM-D478N+D579N was prepared by replacement of the AgeI*/*DraIII DNA fragment of the pET28b-dHis-GST-SPM-D478N by that of the pET28b-dHis-GST-SPM-D579N construct.

For construction of the pEMOC2-ApxIVAΔ629-827 allelic exchange vector, two DNA fragments corresponding to the 5′ and the 3′ flanking regions of the in-frame deletion were amplified from genomic DNA purified from the Czech field isolate Actinobacillus pleuropneumoniae KL2-2000 (biotype 1, serotype 9) using PCR, as follows: the 998-bp DNA fragment upstream of the deletion was amplified from DNA by using the forward primer 5′-TTTGCGGCCGCTTGCGGGCAAAGAAGTTACG-3′ containing the NotI restriction site and the reverse primer 5′-TTTCTCGAGATTTGGCGCATTCACATCGC-3′ containing the XhoI site. Similarly, the 1,004-bp DNA fragment downstream of the deletion was amplified from DNA by using the forward primer 5′-TTTCTCGAGCGCACAATTAATCTAACCGG-3′ containing the XhoI site and reverse primer 5′-TTTGGGCCCAATTTTAAGGTGTCAATATCGC-3′ containing the ApaI site. The PCR products were cut with appropriate restriction enzymes and collectively ligated with the NotI/ApaI-cleaved pEMOC2 vector. All constructs were confirmed by DNA sequence analysis with an ABI Prism 3130XL analyzer (Applied Biosystems, USA) using a BigDye Terminator cycle sequencing kit.

### Protein expression and purification.

All GST-SPM fusion constructs were expressed in E. coli strain BL21(λDE3) transformed with the appropriate plasmid. Exponential 500-ml cultures were grown in a shaking incubator at 37°C in M9 minimal medium supplemented with trace metals, vitamins, and kanamycin (60 μg/ml). For NMR experiments, the cells were grown in M9 medium supplemented with 0.5 g/liter ^15^NH_4_Cl (Cambridge Isotope Laboratories, USA) and 2 g/liter [^13^C]glucose (Cambridge Isotope Laboratories, USA). Expression of proteins was induced by adding 1 mM isopropyl-β-d-thiogalactopyranoside (IPTG) at an optical density at 600 nm (OD_600_) of 0.6 to 0.8, and bacteria were grown for an additional 4 h. The cells were harvested by centrifugation (1,500 × *g* for 15 min), washed in TNE buffer (50 mM Tris-HCl, pH 7.4, 150 mM NaCl, 5 mM EDTA), and disintegrated by sonication in TN buffer (50 mM Tris-HCl, pH 7.4, 150 mM NaCl) at 4°C. The cell extracts were centrifuged at 20,000 × *g* for 30 min, and the supernatants were used for protein purifications.

The purification of the wild-type Ca-SPM was previously described (31). Briefly, the cleared cell lysate was loaded onto Ni­Sepharose 6 Fast Flow beads (GE Healthcare) and washed with TN buffer, and the dHis-GST­SPM protein was eluted with TN buffer supplemented with 500 mM imidazole. The collected fractions were mixed with dithiothreitol (DTT) to a final concentration of 10 mM before the protein solution was dialyzed overnight at 4°C in TN buffer supplemented with 10 mM DTT and 10 mM CaCl_2_. Addition of calcium ions induced a self-processing activity of SPM, resulting in the cleavage of the GST­SPM fusion proteins. The cleaved GST protein was precipitated by incubation of the protein mixture at 70°C for 15 min, and the resulting suspension was centrifuged at 5,000 × *g* for 20 min. The supernatant was loaded onto a PLRP­S reverse-phase column (Agilent Technologies) in buffer containing 50 mM triethylamine (pH 8.5) and 5% acetonitrile, and SPM was eluted from the column by the gradient of acetonitrile (5 to 95%). The SPM fractions were concentrated by rotary vacuum evaporator and loaded onto a Superdex HR 200 gel filtration column (GE Healthcare) equilibrated with TN buffer supplemented with 10 mM CaCl_2_. For purification of SPM-P415A, the cell lysate containing GST-SPM-P415A was loaded onto a glutathione agarose column (Life Technologies), extensively washed with TN buffer, and eluted with TN buffer supplemented with 10 mM l-glutathione (reduced). Collected fractions were pooled, mixed with the purified recombinant tobacco etch virus (TEV) protease (1:20, wt/wt), and dialyzed at 4°C overnight against TN buffer. The protein mixture was incubated at 70°C for 15 min, and the resulting suspension was centrifuged at 5,000 × *g* for 20 min. The supernatant was loaded onto a PLRP­S reverse-phase column (Agilent Technologies) in buffer containing 50 mM triethylamine (pH 8.5) and 5% acetonitrile, and SPM-P415 was eluted from the column by the gradient of acetonitrile (5 to 95%). The collected fractions were concentrated by rotary vacuum evaporator and loaded onto a Superdex HR 200 gel filtration column (GE Healthcare) equilibrated with TN buffer supplemented with 10 mM CaCl_2_. The single (D478A, D478N, D579A, and D579N) and double (D478N D579N) substitution mutants of GST-SPM constructs were purified by Ni­Sepharose 6 Fast Flow column chromatography as described above for the wild-type SPM. The collected fractions were mixed with dithiothreitol (DTT) to a final concentration of 10 mM before overnight dialysis at 4°C in TN buffer. The purity of the proteins was monitored by SDS-polyacrylamide gel electrophoresis (SDS-PAGE), and protein concentrations were determined by Bradford assay (Bio-Rad) using bovine serum albumin as a standard.

### NMR spectroscopy.

NMR spectra were acquired at 30°C on Bruker Avance III 600 MHz spectrometer equipped with triple-resonance (^1^H-^13^C-^15^N) inverse and quadruple-resonance (^1^H-^31^P-^13^C-^15^N) inverse cryoprobes, a Bruker Avance III HD 700 MHz NMR spectrometer equipped with a triple-resonance (^1^H-^13^C-^15^N) cryoprobe optimized for ^13^C detection, a Bruker Avance III HD 850 MHz NMR spectrometer equipped with a triple-resonance (^1^H/^19^F-^13^C-^15^N) inverse cryoprobe, and a Bruker Avance III HD 950 MHz NMR spectrometer equipped with a triple-resonance (^1^H-^13^C-^15^N) inverse cryoprobe. The uniformly ^13^C-^15^N-labeled samples were measured in buffer containing 10 mM Tris-HCl (pH 7.4), 50 mM NaCl, 10 mM CaCl_2_, 10% D_2_O, and 0.1% NaN_3_ at protein concentrations of 0.55 mM and 0.35 mM for Ca-SPM and Ca-SPM-P415A samples, respectively. Two-dimensional (2D) ^1^H-^15^N HSQC, three-dimensional (3D) HNCO, 3D HNCACB, 3D CBCA(CO)NH, and 3D H(C)CH total correlation spectroscopy (TOCSY) spectra measured at 600 MHz and 3D aliphatic ^13^C-edited NOESY and 3D ^15^N-edited NOESY spectra measured at 950 MHz were obtained for the SPM-P415A sample. 3D HNCO, 3D HNCACB, 3D CBCA(CO)NH, 3D ^15^N-edited NOESY, 3D H(C)CH-TOCSY, and 3D aromatic ^13^C-edited NOESY spectra measured at 600 MHz, 2D aromatic ^1^H-^13^C HSQC spectrum measured at 700 MHz, 2D ^1^H-^15^N HSQC, ^15^N *R*_1_, ^15^N *R*_2_, steady-state ^1^H-^15^N NOE enhancement (ssNOE) spectra measured at 850 MHz, and 3D aliphatic ^13^C-edited NOESY spectrum measured at 950 MHz were obtained for the SPM sample. In addition, 2D ^1^H-^15^N HSQC, 3D HN(CO)CA, and 3D HNCA spectra of Ca-SPM were recorded at 10°C at 850 MHz, and 3D HNCACB and 3D CBCA(CO)NH were measured at 10°C at 950 MHz. Sequential assignment of backbone and side chain ^1^H, ^13^C, and ^15^N resonances of SPM was performed by standard triple-resonance NMR techniques (32). All NMR spectra were processed and analyzed with NMRPipe, version 8.7 (33), and Sparky, version 3.115 (T.D. Goddard and D. G. Kneller, University of California, San Francisco, USA). The automated assignment of NOESY spectra and the structure calculation were performed in CYANA, version 3.97 (34).

### Structure calculation of SPM and SPM-P415A.

Torsion angle restraints were determined from chemical shifts by TALOS-N (35) using the ^1^H^N^, ^1^H^α^, ^15^N, 13C′, ^13^C^α^, and ^13^C^β^ resonances. Distance restraints were derived from the automatically assigned NOESY spectra with a manual check of the results. Distance and torsion restraints were used to generate 100 structures in CYANA, version 3.97. Twenty structures with the lowest target functions were used as an input for the molecular dynamics (MD) simulations. In calculations with calcium ions, four Ca^2+^ ions were introduced in CYANA, version 3.97, by defining three 3-Å distance restraints between Ca^2+^ and oxygen OD1 of D419, D421, D423, D462, N464, N468, D499, N501, D503, D521, N523, and D525. The fifth Ca^2+^ ion was introduced into the constructs containing D414, where the distance restraints were to oxygen OD1 of D414, D478, and D579. The Ca^2+^ ions were not restrained in the final refinement during the MD runs.

### MD simulations.

Molecular dynamics (MD) simulations were performed in GROMACS, version 5.0.5 (36, 37) using the force field parameters AMBER99SB-ILDN (38). On average, the systems were solvated in 8,200 explicit TIP3P (39) water molecules and 12 Cl^−^ and 18 (minus two times number of Ca^2+^ ions introduced) Na^+^ ions in a periodic dodecahedron simulation box mimicking the electroneutral system with a salt concentration of 70 mM. The MD step of 1 fs was used in the leapfrog integration scheme. Van der Waals interactions were calculated using a triple-range cutoff scheme, with the updated interaction distance of 1 nm. Electrostatic interactions were calculated by the particle mesh Ewald (PME) method with a Coulomb cutoff of 1 nm and a relative dielectric constant of 1. The temperature and pressure were controlled by a Berendsen thermostat (40) with a coupling constant of 0.1 ps and a Parrinello-Rahman barostat (41) at the constant pressure of 10^5^ Pa with the coupling constant of 1 ps. Simulated annealing of the system was initiated with a relaxation phase of 100 ps, equilibration at 300 K, and subsequent heating to 1,000 K in 50 ps. The temperature was kept constant for 3 ns and then slowly decreased to 0 K over an additional 12 ns. The structure was restrained during MD by experimental distances and dihedral angles with force constants of 1,000 kJ mol^−1 ^nm^−2^ and 350 kJ mol^−1 ^rad^−2^, respectively.

The quality of the structures was analyzed by PROCHECK (42), WHAT IF (43), and CING (44), available as the online service iCING. Ramachandran statistics by program PROCHECK categorized 91.3 and 92.4% residues in the most favored regions, 8.6 and 7.4% in the additional allowed regions, and 0.1 and 0.1% in the generously allowed regions for SPM and SPM-P415A, respectively. No residues were found in the disallowed regions. Secondary structure motifs were identified from the models by the program DSSP (45) with a threshold of 75% of occurrence. The electrostatic potentials were calculated at 303.2 K in APBS (46). The chemical shifts were predicted from calculated structures by SPARTA+ (47).

### Titration of Ca-SPM and Ca-SPM-P415A by EDTA.

An unlabeled SPM or SPM-P415A sample was prepared in the same buffer as used for the structure determination and was dialyzed overnight against a CaCl_2_-free buffer, consisting of 50 mM Tris-HCl (pH 7.4) and 50 mM NaCl; 10% D_2_O and 0.1% NaN_3_ were added to the sample prior to the measurement. The final volume and protein concentration were 0.54 ml and 0.175 mM or 0.52 ml and 0.707 mM for SPM or SPM-P415A, respectively. The titration was monitored by a 1D proton NMR experiment with water suppression using the WATERGATE W5 pulse sequence with gradients in a double echo at 700 or 850 MHz, and the delays for water suppression were 205 or 169 μs. The total number of scans was 192 or 80, and the spectral width was 20 or 30 ppm for SPM or SPM-P415A, respectively. The delay for the interscan was 5 s, and spectra were sampled by 64,000 points. The samples were titrated with the solution of 50 mM EDTA, 50 mM NaCl, and 50 mM Tris-HCl (pH 7.4). The 1D spectra were processed with apodization exponential 1 Hz function and phased.

### ^15^N Relaxation measurements.

The NMR ^15^N relaxation experiments were carried out at 30°C using ^15^N heteronuclear single-quantum coherence (HSQC)-based pulse schemes on a uniformly ^15^N-labeled Ca-SPM (0.33 mM). The temperature was calibrated with a 100% methanol sample, where difference between peaks was set up to 1.518 ppm (48). The following relaxation delays were used in the ^15^N *R*_1_ experiment: 11.2, 78.4, 168.0, 268.8, 380.8, 492.8*, 638.4, 795.2, 985.6, and 1,232.0 ms; the following relaxation delays were used in the ^15^N *R*_2_ experiments: 0, 14.4, 28.8, 43.2, 57.6, 72.0*, 86.4, 100.8, and 115.2 ms. Asterisks denote spectra recorded twice in order to estimate experimental error. Each 2D ^15^N *R*_1_ and ^15^N *R*_2_ experiment was composed of 320 by 2,048 complex points in the indirect ^15^N and direct ^1^H dimensions, respectively, corresponding to respective acquisition times of 61.5 ms and 80.2 ms. Experiments were acquired with 8 scans per free induction decay (FID) and an interscan delay of 2 s. The relaxation rates were obtained by fitting peak intensities to a mono-exponential decay by using Relax (49). The ^1^H-^15^N ssNOE measurements were achieved with a uniformly ^15^N-labeled Ca-SPM (0.6 mM) under a steady-state condition, achieved by a 5-ms ^1^H irradiation with 225 repeats of 200-μs 180° pulses separated by 22.22-μs delays and with a 15-s interscan delay. The recorded spectra consisted of 320 by 2,048 complex points in the indirect ^15^N and direct ^1^H dimensions, respectively, corresponding to respective acquisition times of 57.7 ms and 80.3 ms for a reference and steady-state spectra, which were measured in an interleaved manner. Experiments were acquired with 16 scans per free induction decay (50). Fast backbone motions of Ca-SPM were calculated by a model-free approach (51). The program ROTDIF (52) was used to calculate a rotational diffusion tensor in order to separate the influence of the slow exchange from the effect of the rotational diffusion. The axially symmetric model was used for description of the experimental data.

### CD spectroscopy.

The far-UV circular dichroism (CD) spectra were recorded on a Jasco-815 spectropolarimeter in rectangular quartz Suprasil cells of 1-mm path length (110-QS; Hellma). Protein samples (100 μg/ml) were diluted in 5 mM Tris-HCl (pH 7.4) plus 50 mM NaCl in the absence or presence of 10 mM CaCl_2_ and measured for wavelengths from 195 to 280 nm at 25°C. Two spectrum accumulations with standard instrument sensitivity and scanning speed of 10 nm/min with response time of 16 ms were acquired. The spectra of the buffers were subtracted from the protein spectra, and mean molar ellipticity (θ) was expressed in degrees per square centimeter per decimole.

### Thermal stability assay.

Thermal stability of SPM proteins was performed by nano-differential scanning fluorimetry (nanoDSF) using a Prometheus NT.48 instrument (NanoTemper Technologies, Munich, Germany). Ca-SPM and Ca-SPM-P415A were dialyzed overnight at 4°C against TN buffer, and the freshly prepared protein samples were supplemented with increasing concentrations of calcium ions (0 to 10 mM CaCl_2_) before 10 μl was used for filling the Prometheus NT.48 high-sensitivity capillaries (NanoTemper Technologies). The measurements were conducted from 20 to 95°C (with a temperature ramp of 2.5°C/min) under constant monitoring of tryptophan fluorescence at 350 and 330 nm. The melting temperature (*T_m_*) values, corresponding to the inflection points of the unfolding curve, were determined via the first derivative of the curve.

### Construction of the *apxIVA* mutant strain of A. pleuropneumoniae.

In-frame deletion in the *apxIVA* gene (codons 629 to 827) was performed on chromosome of the Czech field isolate Actinobacillus pleuropneumoniae KL2-2000 (biotype 1, serotype 9) by homologous recombination using a pEMOC2 allelic exchange vector as previously described (53). Briefly, A. pleuropneumoniae cells grown in a 5% CO_2_ atmosphere at 37°C on Bacto tryptic soy broth agar (BD Biosciences) supplemented with 10 μg/ml β-NAD (NAD; Sigma) were mated on fresh agar plates supplemented with 10 μg/ml NAD, 1 mM diaminopimelic acid (DAPA; Sigma), and 10 mM MgSO_4_ with E. coli β2155 grown on LB agar supplemented with 1 mM DAPA and 25 μg/ml chloramphenicol (Cm) before being transformed with the pEMOC2-ApxIVAΔ629-827 plasmid construct. After 4 h at 37°C, the A. pleuropneumoniae transconjugants were selected on Bacto tryptic soy broth agar plates supplemented with 10 μg/ml NAD and 5 μg/ml chloramphenicol and incubated in 5% CO_2_ for 24 h at 37°C. For sucrose counterselection, the single Cm^r^ colonies were inoculated into salt-free LB broth supplemented with 10% sucrose, 10% horse serum, and 10 μg/ml NAD, incubated with shaking at 37°C for 2 h, and plated on salt-free LB broth supplemented with 10% sucrose, 10% horse serum, and 10 μg/ml NAD. Sucrose-resistant colonies were screened for the truncated variant of the *apxIVA* gene by a colony PCR and restriction analysis of the resulting PCR products (XhoI).

### Animal experiments.

The animal experiments were carried out according to the guidelines of the Animal Care Act (no. 246/1992 Coll.) of the Czech Republic, approved by the Animal Welfare Commission of the Ministry of Agriculture of the Czech Republic, and conducted in accredited barrier-type stables (accreditation certificate no. 5843/2007-10001). Thirty 4-weak-old Large White pigs with body weights of 7 to 10 kg were purchased from a porcine reproductive and respiratory syndrome virus-free and A. pleuropneumoniae infection-free herd (as tested by serological reaction in enzyme-linked immunosorbent assays [ELISAs]) and randomly assigned to five groups, each containing six animals. Pigs were regularly monitored throughout the housing period at least three times per day.

### Experimental infection.

The virulence of A. pleuropneumoniae strains was assessed in an intranasal infection model as described previously (54). Briefly, pigs were anesthetized by intravenous injection of ketamine (4 mg/kg of body weight) and xylazine (2 mg/kg) and intranasally administered 4 ml (2 ml per nostril) of phosphate-buffered saline (PBS) solution (control group) or infected with two doses of 2 ml of bacterial suspension corresponding to an infection dose of 10^6^ and 10^9^ CFU per animal. The CFU number was measured spectrophotometrically in the bacterial culture grown at 37°C in brain heart infusion broth (HiMedia, India) supplemented with 10 μg/ml NAD, in which an optical density at 550 nm (OD_550_) of 1.0 corresponded to 5 × 10^9^ CFU/ml. Pigs were monitored for 3 days after infection, and the A. pleuropneumoniae-related clinical signs (respiratory rate, dyspnea, coughing, anorexia, and lethargy) were recorded. At −24, 0, 24, 48, and 72 h postinfection, body temperature was measured in the rectum. Blood samples were collected by jugular venipuncture into heparinized tubes (30 IU/ml) at −24, 24, and 72 h postinfection and immediately used for cytological examination. Cell counts were determined by a BC-2800Vet Auto Hematology Analyzer (Mindray, China). All pigs were examined postmortem for macroscopic pathological changes and for the presence of bacteria. The lung score was determined as the extent of lung tissue damage (lung lesions) according to the percentages for the following anatomical segments: left apical lobe, 8%; right cranial lobe, 12%; left cardiac lobe, 8%; right medium lobe, 12%; left diaphragmatic (caudal) lobe, 25%; right diaphragmatic (caudal) lobe, 30%; and accessorius lobe, 5%. For bacterial examination, nasal and tracheal swabs were inoculated on Columbia agar supplemented with 5% sheep blood with a streak of Staphylococcus aureus (as a source of NAD) and cultivated aerobically at 36°C for 18 h.

### Data availability.

NMR chemical shifts and distance restraints have been deposited in the Biological Magnetic Resonance Bank (BMRB) under entries 28058 (http://www.bmrb.wisc.edu/data_library/summary/index.php?bmrbId=28058) and 34424 (http://www.bmrb.wisc.edu/data_library/summary/index.php?bmrbId=34424) for Ca-SPM and Ca-SPM-P415A, respectively. Structural coordinates of Ca-SPM and Ca-SPM-P415A have been deposited in the Protein Data Bank under accession codes PDB 6SJW (https://www.rcsb.org/structure/6sjw) and PDB 6SJX (https://www.rcsb.org/structure/6sjx), respectively. All other data are available from the authors upon request.

10.1128/mBio.00226-20.7 TABLE S1 Virulence of the wild-type and the mutant strains of A. pleuropneumoniae serotype 9 in a pig respiratory challenge model. Download Table S1, DOCX file, 0.01 MB. Copyright © 2020 Kuban et al. 2020 Kuban et al. This content is distributed under the terms of the Creative Commons Attribution 4.0 International license.
