# Supplementary material for: Structural Basis of Ca2+-Dependent Self-Processing Activity of Repeat-in-Toxin Proteins
Source: mBio. 2020 Mar 17;11(2):e00226-20. doi: 10.1128/mBio.00226-20 (PMC7078468; doi:10.1128/mBio.00226-20)
Supplement: TABLE S1 [file mBio.00226-20-st001.docx]

**Supplementary Table 1.**

|  | challenge dose (CFU) | No. of animals with reisolation of challenged strains | pleuritis | body temperature (>40.5 °C) |
| --- | --- | --- | --- | --- |
| control | 0 | 0/6 | 0/6 | 0/6 |
| A.p. | low (4×10^6^) | 3/6 | 0/6 | 3/6 |
|  | high (6×10^9^) | 6/6 | 2/6^a^ | 1/2^a^ |
| A.p.-ΔSPM | low (8×10^6^) | 5/6 | 1/6 | 4/6 |
|  | high (2×10^10^) | 6/6 | 2/6^b^ | 1/1^b^ |

^a^4 out of 6 piglets died within 24 h post infection, 2 piglets were euthanized at 24 h post infection

^b^5 out of 6 piglets died within 24 h post infection, 1 piglet was euthanized at 24 h post infection
